# Intrauterine Programming of Cardiovascular Diseases in Maternal Diabetes

**DOI:** 10.3389/fphys.2021.760251

**Published:** 2021-11-03

**Authors:** Romina Higa, María Laura Leonardi, Alicia Jawerbaum

**Affiliations:** ^1^Facultad de Medicina, Universidad de Buenos Aires, Buenos Aires, Argentina; ^2^Laboratory of Reproduction and Metabolism, CONICET-Universidad de Buenos Aires, CEFYBO, Buenos Aires, Argentina

**Keywords:** heart, offspring, animal model, humans, diabetes mellitus, intrauterine programming, pathways

## Abstract

Maternal diabetes is a prevalent pathology that increases the risk of cardiovascular diseases in the offspring, the heart being one of the main target organs affected from the fetal stage until the adult life. Metabolic, pro-oxidant, and proinflammatory alterations in the fetal heart constitute the first steps in the adverse fetal programming of cardiovascular disease in the context of maternal diabetes. This review discusses both human and experimental studies addressing putative mechanisms involved in this fetal programming of heart damage in maternal diabetes. These include cardiac epigenetic changes, alterations in cardiac carbohydrate and lipid metabolism, damaging effects caused by a pro-oxidant and proinflammatory environment, alterations in the cardiac extracellular matrix remodeling, and specific signaling pathways. Putative actions to prevent cardiovascular impairments in the offspring of mothers with diabetes are also discussed.

## Introduction

Cardiovascular diseases are increasing at alarming rates in both developed and developing countries (Balakumar et al., 2016; Bhatnagar et al., 2016). Although lifestyle choices and genetic predisposition are the main contributors to cardiometabolic diseases, growing evidence indicates that *in utero* exposure to adverse environmental conditions leads the developing offspring to have numerous risk factors, which may have an impact later in life. The concept of developmental programming was first introduced more than 20 years ago by Dr. David Barker, who investigated the association between low birth weight and increased risk of coronary disease in adult life (Barker, 1998). This phenomenon describes the process by which a stimulus or insult during critical periods of growth and development has lasting effects on the structure or function of tissues and organ systems, which will in turn influence changes in body structure and function permanently. Programming occurs because there is a deregulation of the endocrine or metabolic function, or a failure in the development, growth, or interaction of tissues and organ systems of the body. These alterations involve disruptions in gene expression, cell differentiation, proliferation, communication, and/or signaling during critical periods of fetal life and infancy caused by adverse environmental influences. In particular, the risk of fetal programming of metabolic and cardiovascular diseases is well-known to be increased by diabetes in pregnancy (Pandey et al., 2015; Lohse et al., 2018).

Diabetes can be diagnosed before pregnancy (pregestational type 1 or type 2 diabetes) or have its onset during pregnancy (gestational diabetes mellitus, GDM). All types of diabetes have been shown to induce an adverse fetal programming (Poston, 2010). Epidemiological studies performed in both developed and developing countries have shown an increase in the prevalence and incidence of type 2 diabetes and GDM over the years (Trujillo et al., 2015; Zhu and Zhang, 2016; Hills et al., 2018; Najafi et al., 2019; Gorban de Lapertosa et al., 2021). This review provides information on studies of both human and experimental models addressing putative mechanisms involved in the fetal programming of heart damage in maternal diabetes. Shedding light on these mechanisms would provide tools for future studies in the prevention of cardiovascular disease in offspring exposed to maternal diabetes.

## Human Studies

Several studies have shown that children and adolescents exposed to maternal diabetes *in utero* have significantly higher systolic and mean arterial blood pressures (Cho et al., 2000; Lu et al., 2019; Pathirana et al., 2019). In addition, a correlation has been found between GDM and the rate of cardiovascular disease-related hospitalizations of the offspring up to 18years (Leybovitz-Haleluya et al., 2018). A recent study has shown that fetuses from diabetic gestation present an increase in the left ventricular mass and wall thickness, which persist into late infancy (Do et al., 2019). The follow up of this longitudinal study in childhood has shown that these alterations persist, with the offspring from diabetic mothers showing higher aortic and ventricular stiffness (Do et al., 2021). Further, a 40-year follow-up study in Denmark has shown that offspring of mothers with diabetes have 29% increased overall rate of early onset of cardiovascular heart disease (heart failure, hypertensive disease, deep vein thrombosis, and pulmonary embolism; Yu et al., 2019).

Cardiovascular diseases associated with inflammatory processes, as the ones related to diabetes, show increased endothelial cell signal transductions, which favor leukocyte migration by endothelial cell adhesion molecules (Endemann and Schiffrin, 2004). Among these molecules, vascular cell adhesion molecule-1 (VCAM-1) plays important roles in the embryonic development of the cardiovascular system and in cardiovascular diseases (Cook-Mills et al., 2011). In this regards, it has been found that children born to mothers with diabetes during pregnancy have increased levels of VCAM-1 and other markers of endothelial activation like E-selectin (Manderson et al., 2002; West et al., 2011). A study of 21 healthy non-Hispanic White children between 8 and 12years old exposed to GDM *in utero* reported that, in addition to higher levels of VCAM-1, these children showed an increase in cardiometabolic risk factors, including higher body mass index *z* scores, higher waist circumference and higher levels of triglycerides, low-density lipoprotein-cholesterol, and leptin compared to unexposed children (West et al., 2011). The latter is important because increased levels of leptin may stimulate oxidative stress, inflammatory reactions, atherogenesis, and thrombosis and thus promote endothelial dysfunction, arterial stiffness, and development and vulnerability of atherosclerotic plaques (Katsiki et al., 2018).

Regarding proposed mechanisms of programming of cardiovascular alterations by maternal diabetes, the main protagonist is epigenetics. Epigenetics can be defined as heritable changes that modify gene expression without altering the DNA sequence itself. Epigenomes are sensitive to their environment and are affected by specific environmental cues, being a plausible mechanism that conditions developmental origin of health and disease in later life. Epigenetic responses during critical windows of fetal development alter gene expression in specific organs or tissues, conditioning their developing and immature functionality and resulting in changes in mature tissues and organs. These responses can either protect the organism from or predispose the organism to disease development in later life. Epigenetic modifications include DNA methylation. DNA methylation involves the covalent addition of a methyl group to the cytosine base in CpG dinucleotide islands of regulatory sites of gene promoter regions that regulate gene expression. This pattern of DNA methylation is highly susceptible to abnormal modifications during adverse gestation and neonatal development (Zhou et al., 2011). In this regard, a genome-wide methylation analysis performed in DNA obtained from peripheral blood mononuclear cells from healthy non-Hispanic White children exposed to maternal GDM during intrauterine life showed 84 genes with differentially methylated regions (West et al., 2013). This study also showed that several of the top 10 genes ranked by statistical significance, such as *Natriuretic Peptide Receptor* 1 (*NPR1*; related to blood pressure homeostasis), *panthothenate kinase* (*PANK1*; a critical enzyme in the synthesis of coenzyme A), *SCAN domain-containing protein 1* (*SCAND1*; which encodes a cofactor that interacts with transcription factor regulators of genes involved in lipid metabolism), and *GJA4* (which encodes connexin 37, a component of gap junction channels involved in intercellular communication) were associated with cardiovascular risk (West et al., 2013). Alterations in these genes have been found to be related to hypertensive diseases, altered glucose and lipid metabolism, and myocardial physiology and may thus be related to adverse programming of cardiovascular disease in offspring exposed to maternal diabetes (Babb and Bowen, 2003; Pitzalis et al., 2003; Leu et al., 2011).

Other molecular epigenetic mechanisms that may play a key role in epigenetic inheritance and aberrant development of cardiovascular disease in later life include histone modifications and small non-coding RNAs, including microRNAs (also called miRNAs; Watson et al., 2019).

For example, a study of whole peripheral blood from children aged 3–11years descending from GDM pregnancies showed that these children present an altered miRNA expression profile (Hromadnikova et al., 2020). The study also showed that a large group of the genes affected by the altered miRNA expression is involved in ontologies of diabetes/cardiovascular/cerebrovascular diseases (Hromadnikova et al., 2020). Moreover, clinical examination of these children indicated that they present an increased incidence of valve problems or heart defects (Hromadnikova et al., 2020).

Studies in human umbilical vein endothelial cells (HUVECs) are an excellent model for the study of vascular endothelium properties and the main biological pathways involved in endothelium function.

Studies in primary culture of HUVECs and human placental microvascular endothelial cells from GDM pregnancies have shown the dysregulation of adenosine metabolism (Westermeier et al., 2011, 2015). Adenosine metabolism, which plays an important role in the methylation cycle, is a proposed mechanism of epigenetic regulation in endothelial cells involved in the cardio-metabolic fetal programming by GDM (Silva et al., 2020).

Another study performed in HUVECs from GDM pregnancies reported a reduction in the expression of histone methyltransferase *enhancer of zester homolog 2 – β* (*EZH2β*) related to an increase in the expression of miR-101, a microRNA that targets *EZH2β* (Kuzmichev et al., 2002). *EZH2β* is part of a multisubunit complex that initiates and maintains the trimethylation of histone H3 on lysine 27 (H3K27me3), an epigenetic mark associated with heterochromatin formation and gene silencing (Cao et al., 2002), and thus probably related to the regulation of this epigenetic mark in endothelial cells from GDM offspring.

The knowledge regarding the mechanisms of programming of cardiovascular disease in offspring from human patients with diabetes is limited because of the difficulty in performing human intervention studies. Thus, the studies of maternal diabetes in animal models are relevant as they allow us to better understand the developmental origins of disease, moving our understanding from associative studies to mechanistic insights into disease causation.

## Animal Studies

Among animal studies, those performed in rodent models are useful because the order and timing of fetal organ development are similar to those in humans. Moreover, the ability to control various aspects of pregnancy and the pre- and post-natal environment makes rodent models as attractive experimental systems to examine how maternal diabetes during pregnancy programs the physiology of organ systems and increases the risk of cardiometabolic disease in the offspring.

Experimental models of diabetes in pregnancy can have a genetic origin or be obtained by chemical induction with drugs such as streptozotocin (STZ), which, at the appropriate dose, acts by selectively destroying pancreatic cells, leading to insulin deficiency and hyperglycemia in different animals (Jawerbaum and White, 2010).

Regarding the mechanisms underlying the programming of cardiovascular diseases, studies in animal models can be divided into those focused on (1) epigenetic mechanisms, (2) altered pathways of cellular metabolism, (3) alterations induced by a pro-oxidant/proinflammatory environment, and (4) alterations in extracellular matrix remodeling and intercellular communication.

### Animal Studies Focused on Cardiovascular Epigenetic Mechanisms

Animal studies focused on epigenetic mechanisms may improve our understanding of how epigenetic gene transcriptional regulation responds to an altered gestational milieu to influence the developmental origin of health and disease.

As an example, a study of the neonatal heart in a mouse model of diabetes showed a 10-fold increase in the DNA methylation of gene promoter regions of many important cardiac genes (Lister et al., 2013). In another study, rat neonatal hearts exposed to maternal diabetes showed altered gene-activating (H3Ac, H3K4me3) and gene-suppressive (H3K27me3) histone marks (Upadhyaya et al., 2017). In this study, chromatin-immunoprecipitation-sequencing and bioinformatics identified that the promoters of two functionally related genes, *heat shock protein 1a1* (*Hspa1a*) and *Hspa1b*, showed enriched H3K4me3 peaks (Upadhyaya et al., 2017). Hspa1a has been proposed as an independent prognostic marker of heart failure (Jenei et al., 2013). This suggests that Hspa1a may be a relevant marker linking maternal diabetes and offspring heart failure.

In a rat model of diabetes in pregnancy induced by STZ administration on day 12 of gestation, cardiomyocytes from 6-week-old offspring showed an increase in global DNA methylation status, together with an increase in the cardiac expression of DNA methyltransferase 3A and reduced expression of the silent information regulator 1 (Sirt1), a class III histone deacetylase (Chen et al., 2019). This altered expression of Sirt1 was reversed *ex vivo* with a DNA-methylation inhibitor and *in vivo* with the antioxidant N-acetyl-cysteine, suggesting that Sirt1 expression in the heart of offspring from dams with diabetes is epigenetically regulated and affected by an oxidative environment (Chen et al., 2019). Sirt1 functions as a regulator of acetylation, which is important to maintain cardiac mitochondrial integrity and normal myocardium development (Planavila et al., 2012). One of Sirt1 targets genes is the transcription factor forkhead box protein O1 (FoxO1), which, in the cardiovascular system, participates in myocardial metabolic stress adaptation, oxidative stress, endothelial dysfunction, and other processes related to inflammation and apoptosis (Kandula et al., 2016). FoxO1 deacetylation by Sirt1 induces the attenuation of its ability to bind DNA and favors its phosphorylation and consequently its inactivation. In line with this, in a rat model of mild diabetes, the heart of male adult offspring was found to show an increase in active FoxO1 (Musikant et al., 2019).

Another putative epigenetic mechanism involved in the programming of cardiovascular diseases involves biogenesis of miRNAs which can be delivered by exosomes. Exosomes are nanovesicles that contain intact and functional mRNAs, miRNAs and proteins, which function as pivotal mediators of cell-cell communication among neighboring or distant cells and play a key role in multiple physiological or pathological processes (Greening et al., 2017).

It has been demonstrated that, during pregnancy, both feto-maternal and maternal-fetal exosomal trafficking can occur, and that exosomes are able to cross placental barriers and reach fetal tissues (Sheller-Miller et al., 2019; Czernek and Düchler, 2020). Although the precise mechanism has not yet been completely clarified, *in vitro* studies have shown that endothelial cells absorb placental-derived exosomes through endocytosis (Tong et al., 2017). A study by Shi et al. of serum exosomes isolated from pregnant mice with STZ-induced diabetes showed that these vesicles present differences in the expression of more than 200 miRNAs (Shi et al., 2017), some of which, including miR-133, miR-30, miR-99, and miR-23, are involved in cardiac development and cardiovascular diseases (Bang et al., 2012; Izarra et al., 2017; Yin and Yang, 2019). These authors also found that serum exosomes isolated from these diabetic pregnant mice, fluorescently labeled for tracking and then injected into normal pregnant mice *via* the tail vein, were able to cross the placental barrier and infiltrate into embryonic tissues, including the heart (Shi et al., 2017). Finally, these authors showed that the injections of these exosomes increased embryonic heart malformations and induced alterations in the cardiac systolic function in the morphologically normal fetus (Shi et al., 2017), highlighting the role of maternal exosome-derived microRNAs in the induction of fetal cardiovascular defects.

### Animal Studies Focused on Altered Pathways of Cardiac Cellular Metabolism

Key pathways of cellular metabolism have been shown to be altered in cardiac cells of offspring from animal models of diabetes. A transcriptomic study performed in neonatal heart from dams treated with a combination of high-fat diet and STZ at day 14 of pregnancy, showed many changes in key genes of metabolic cardiac pathways. Among them, a downregulation of *fibroblast growth factor* (*FGF*), which in turn downregulates PI3K/AKT pathway activation that lead to increased *glycogen synthase kinase 3 β* (*GSK3β*) and peroxisome *proliferator-activated receptor gamma coactivator alpha* (*PGC1α*; Preston et al., 2020). PI3K/AKT is an insulin sensitive pathway that modulates metabolic pathways as gluconeogenesis (modulated by GSK3β) and mitochondrial function and biogenesis (modulated by PGC1α), between others. These changes would contribute to an increased translation of mitochondrial proteins, mitochondrial biogenesis, and gluconeogenesis.

Another important pathway of cellular metabolism is regulated by the mechanistic target of rapamycin (mTOR), a cellular sensor for energy metabolism and nutrient availability that controls cellular growth and metabolism. mTOR forms two different complexes (mTORC1 and mTORC2), which differ in downstream signaling pathways and function (Saxton and Sabatini, 2017). Activated mTORC1 plays a central role in the control of cell growth and proliferation, while mTORC2 plays a key role in cell survival, metabolism, proliferation, and cytoskeleton organization (Saxton and Sabatini, 2017). In a study of a rat model of STZ-induced mild diabetes, the heart of adult offspring showed a reduction in mTOR protein levels and in the phosphorylation of serum- and glucocorticoid-inducible kinase 1 (SGK1), a downstream target of the mTORC2 pathway, which phosphorylates FoxO1 (Musikant et al., 2019). Phosphorylation of the FoxO family by SGK1 induces its nuclear exclusion and inactivation, providing another possible mechanism explaining FoxO1 increased activity (Brunet et al., 2001; Musikant et al., 2019). Interestingly, in a study in which a diet enriched in fish oil was administered to dams with STZ-induced diabetes, the offspring’s neonatal heart showed an increase in the reduced phosphorylation of mTOR and AKT, both in Ser-473 (phosphorylation induced by mTORC2) and in Thr-308 (phosphorylation activated by growth factors pathways; Nasu-Kawaharada et al., 2013).

In the offspring heart of an obesity model with hyperglycemia and hyperinsulinemia, Zhang et al. found that the mTORC1 signaling pathway was increased. These authors also found that a maternal treatment with the antioxidant N-acetyl-cysteine during pregnancy normalized the altered mTOR signaling in the offspring heart (Zhang et al., 2021).

Regarding lipid metabolism, in maternal diabetes, insulin concentration, and/or signaling deficiency may lead to an excess of metabolic substrates and impairments in maternal lipid metabolism. In addition, diabetes in pregnancy frequently induces dyslipemia in the mother (Herrera and Ortega-Senovilla, 2010). We have previously reviewed the importance of lipids in the programming of metabolic and cardiovascular alterations in the offspring (Higa and Jawerbaum, 2013).

In an experimental model of high-fat and high-cholesterol diet during pregnancy and lactation, which induces dyslipemia, glucose intolerance, and reduced insulin sensitivity, male offspring showed increased blood pressure, impaired vascular reactivity, and lower response to endothelium-dependent vasorelaxation (Guimarães et al., 2020). Interestingly, in that study, an administration of a specific strain of the probiotic *Lactiplantibacillus plantarum* improved the lipid profile and insulin resistance and restored the dysbiotic gut microbiota in dams, reducing blood pressure and recovering the vascular function in the adult offspring (Guimarães et al., 2020). In another study in a STZ-induced experimental model of mild diabetes, 5-month-old offspring showed increased glycemia, insulinemia, and triglyceridemia (Capobianco et al., 2015). Besides, a sex-dependent lipid accumulation was observed in their heart as female offspring showed increased levels of free fatty acids, cholesterol, and phospholipids and male offspring showed increased levels of triglycerides (Capobianco et al., 2015). These results are relevant because an increase in lipid accumulation in the heart has been related to impaired mitochondrial metabolism and dynamics and increased oxidative stress, cardiomyocyte apoptosis, myocardial fibrosis, and contractile dysfunction (McGavock et al., 2006; Wende and Abel, 2010; Elezaby et al., 2015). Although the mechanisms of sex-dependent lipid overaccumulation in the heart are unknown, they may be due to changes in lipid metabolism, which are in turn dependent on multiple factors, including the sex-dependent impact of hormones, mitochondrial function, and genetic and epigenetic differences (Gabory et al., 2009; Vijay et al., 2015; Regitz-Zagrosek and Kararigas, 2017).

The intrauterine programming of cardiovascular impairments and the regulation of peroxisome proliferator activated receptor (PPAR)-dependent pathways have also been found to be sex-dependent (Benz et al., 2012; Regitz-Zagrosek and Kararigas, 2017). In experimental models of STZ-induced diabetes, PPARα, a nuclear receptor highly involved in heart metabolic processes and lipid metabolism, has been found increased in the heart of both males and females at the neonatal stage but only in that of males at the prepubertal stage (Higa et al., 2017). An increased level of PPARα is a marker of metabolic substrate utilization as this nuclear receptor regulates multiple genes of lipid catabolism (Lefebvre et al., 2006). Oleic acid, present at high concentrations in olive oil, is a natural agonist that interacts with the ligand-binding domain of the PPAR and leads to its activation (Xu et al., 1999). Interestingly, a maternal olive oil-supplemented diet administered during diabetic rat pregnancy prevents lipid overaccumulation in the offspring’s heart, probably as a consequence of improving intrauterine metabolic homeostasis (Capobianco et al., 2015). The PPARα gene was susceptible to epigenetic modification in the livers of offspring of dams fed a low-protein diet (Lillycrop et al., 2005) and PPAR expression in the livers of offspring from an animal model of gestational diabetes was found to be modulated by different miRNAs (Fornes et al., 2018). However, further research in needed to elucidate whether maternal diabetes also induces epigenetic modifications of the PPARα gene in the offspring’s heart.

In an animal model of diabetes induced by STZ administration during pregnancy, results showed diastolic dysfunction in the offspring at the neonatal stage (Mdaki et al., 2016) and impaired mitochondrial dynamics in neonatal cardiomyocytes (Larsen et al., 2019). The term “mitochondrial dynamics” refers to the coordinated cycles of fission and fusion occurring in these organelles, which are important to maintain their shape, distribution and size. Mitochondrial dynamics plays an important role in both cardiac development and long-term heart health (Dorn et al., 2015). Interestingly, a study performed in cardiomyocytes of neonates from rats with diabetes showed 50% reduction in fusion events and 30% reduction in fission events, as well as a pro-fission imbalance in the ratio of these events and a higher number of fragmented mitochondrial morphology (Larsen et al., 2019).

Alterations in mitochondrial function lead to an increase in reactive oxygen species and thus to an increase in oxidative stress (Peoples et al., 2019), which in turn plays a key role in the programming of cardiovascular alterations (Gupta et al., 2004; Rueda-Clausen et al., 2012).

### Animal Studies Focused on Cardiovascular Alterations Induced by a Pro-oxidant/Proinflammatory Environment

In cardiac tissues, a major source of reactive oxygen species under both physiological and pathological conditions is NADPH oxidases (NOX; Lassegue et al., 2012). In a study performed in a rat model of diabetes in pregnancy induced by STZ administration on day 12 of gestation, the heart of the 6-week-old offspring showed an increase in NOX1 and NOX2 together with an increase in reactive oxygen species in the left ventricle (Chen et al., 2019), and the heart of adult offspring still showed an increase in NOX2 expression (Zhang et al., 2018). Maternal treatment with N-acetyl-cysteine prevents the increased NOX2 transcript levels in the offspring heart from a model of obesity and diabetes (Zhang et al., 2021).

In another study in a model of mild diabetes obtained by STZ administration, the offspring’s heart at neonatal stage showed an increase in nitrated proteins, which evidences protein damage by peroxynitrites (Higa et al., 2017), potent oxidant agents generated as the product of nitric oxide and superoxide anion reaction. In that study, neonatal glycemia of offspring exposed or not to maternal diabetes was similar, indicating that peroxynitrite-induced damage in the offspring’s heart occurs in the absence of alterations in neonatal glycemia. However, at a prepubertal stage, glycemia was increased only in male offspring from rats with diabetes (Higa et al., 2017).

Lipids can also be the target of reactive oxygen species. In an experimental model of STZ-induced diabetes, sex-dependent differences were found related to lipoperoxidation, as only neonatal hearts of male offspring showed an increase in thiobarbituric acid reactive substances (TBARS), which are formed as a byproduct of lipid peroxidation (Higa et al., 2017). At the fetal stage, lipoperoxidation has also been observed only in the hearts of male fetuses, suggesting changes during fetal development that persist into adult life (Kurtz et al., 2014).

Regarding maternal treatments that have an impact in modulating the pro-oxidant intrauterine environment, at our lab, we observed that a maternal treatment with an olive oil-supplemented diet administered during pregnancy was able to prevent the increased pro-oxidant markers as well as apoptosis in the heart of 21-day-old offspring of diabetic rats (Roberti et al., 2020). At our laboratory, we have also evaluated a maternal treatment with the mitochondrial antioxidant idebenone in a model of mild diabetes during pregnancy to address the role of oxidative stress and mitochondrial dysfunction in the programming of cardiac alterations and observed that this treatment prevents the increase in markers of oxidative damage in the heart of offspring from diabetic rats at a prepubertal stage (Higa et al., 2017). These results highlight the relevance of mitochondrial-related reactive oxygen species in the programming of heart alterations by maternal diabetes.

### Animal Studies Focused on Alterations in the Cardiac Extracellular Matrix and Intercellular Communication

The cardiac extracellular matrix (ECM) is a complex and dynamic structure that forms a three-dimensional network in the cardiac interstitium. It provides structural support for several distinct cell types, contains growth factors and cellular adhesion proteins, and integrates extracellular signals and cellular responses (Frangogiannis, 2017). In cardiac pathological conditions, ECM synthesis and chemical composition are subject to changes under different environmental stimuli that distort the architecture of the matrix network, modulating the proliferation, migration and activation of cardiac fibroblasts, thus playing a major role in the development of cardiac diseases (Li et al., 2018). During organogenesis, mouse embryo cultures in hyperglycemic conditions have been found to show an increased expression of cardiac fibronectin and transforming growth factor β-1 (TGFβ-1; Smoak, 2004), a fibrogenic growth factor that mediates ECM remodeling. In the male offspring heart from a mouse model of obesity and diabetes, Zhang et al. found an increased expression of TGFβ-1 together with myocardial fibrosis and left ventricular structural alterations. These authors also found that all these programmed alterations were prevented by a maternal treatment with N-acetyl-cysteine during pregnancy (Zhang et al., 2021). Several downstream actions of TGF-β1 can be mediated by connective tissue growth factor (CTGF), a secreted multifunctional protein also involved in the regulation of ECM deposition during development as well as during pathological conditions (Frangogiannis, 2012). A study in an animal model of STZ-induced mild diabetes showed an increase in CTGF levels in the hearts of neonates (Higa et al., 2017). In the heart, CTGF has been found to be epigenetically regulated by microRNA-133 and -30 (Duisters et al., 2009), pointing to these miRNAs as putative epigenetic marks that may be involved in the intrauterine programming of CTGF upregulation in the offspring’s heart as they have also been found increased in serum exosomes isolated from pregnant dams with diabetes (Shi et al., 2017).

The balance between ECM synthesis and degradation is of crucial relevance in maintaining cardiac structural integrity. In this context, it is important to mention the role of matrix metalloproteinases (MMPs), which are proteolytic enzymes able to degrade ECM components. Expression and/or activity of MMPs are upregulated by the pro-oxidant/proinflammatory environment. In particular, MMP-9 expression has been found increased in hearts of neonates from diabetic rats (Higa et al., 2017). Interestingly, alterations in these markers of ECM remodeling persist and are sex-dependent at a prepubertal stage of development. At 21days old, only males show an increased expression of MMP-9, possibly related to increased markers of pro-oxidant/proinflammatory processes, which are observed only in the heart of male fetuses and 21-day-old male offspring (Kurtz et al., 2014; Higa et al., 2017; Roberti et al., 2020). Indeed, the stimulation of several fibrogenic pathways by diabetes is related to the generation of reactive oxygen species and the induction of secretion of pro-inflammatory cytokines and chemokines (Tuleta and Frangogiannis, 2021). Moreover, maternal treatment with an olive oil-supplemented diet administered during pregnancy, which has been found to prevent cardiac pro-oxidant/proinflammatory processes in the offspring, is also able to prevent the increased expression of CTGF and deposition of collagen IV and fibronectin in the heart of 21-day-old offspring of diabetic rats (Roberti et al., 2020).

FoxO1 is a TGFβ-1 downstream crucial player in cardiac fibroblast conversion into cardiac myofibroblasts, which, under pathological conditions such as diabetes mellitus, synthesizes and secretes high amounts of ECM proteins (Vivar et al., 2021). Male adult offspring from rats with mild diabetes show an increase in cardiac active FoxO1, together with an increase in the mRNA levels of its target genes in the heart, *Mmp-2* and *Ctgf*, and in collagen deposition and fibrosis (Musikant et al., 2019). These offspring’s hearts also show reduced expression of connexin 43, a target of matrix metalloproteinase 2 and main component of the gap junctions and hemichannels in myocytes (Musikant et al., 2019). As connexin 43 is a key factor in electrical coupling and its altered expression alters normal impulse propagation (van Rijen et al., 2006), its reduced levels in the offspring’s heart is likely related to cardiac dysfunction. These alterations were found concomitantly with increased markers of cardiomyopathy and increased cardiovascular risk factors as glycemia, triglyceridemia, and insulinemia (Musikant et al., 2019). Together, these results suggest an important role of FoxO1 activation in the cardiac alterations related to cardiac ECM remodeling induced by intrauterine programming in maternal diabetes (Musikant et al., 2019).

## Conclusion

**Figure 1:**
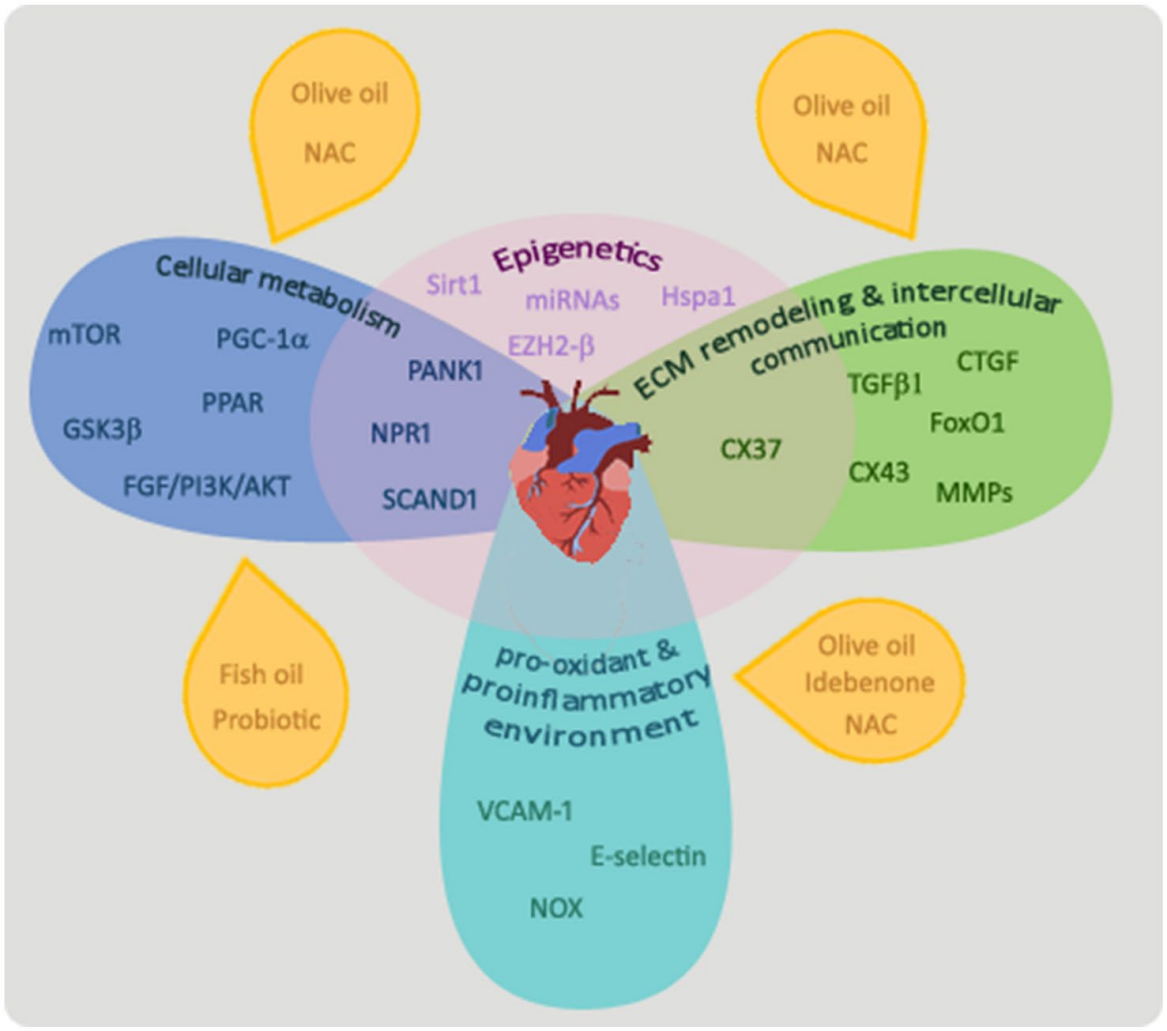
Mechanisms involved in the programming of cardiovascular alterations in offspring by maternal diabetes. Main players that regulate cellular metabolism in the heart of offspring exposed to diabetes *in utero* are mTOR, PPAR, PANK1, PGC1α, GDK3β, NPR1, SCAND1, and the FGF/PI3K/AKT pathway. Maternal treatments with probiotics or a diet enriched in olive or fish oil or N-acetyl-cysteine have been shown to ameliorate cardiac cell metabolism of offspring from diabetic dams. TGFβ-1, CTGF, FoxO1, matrix metalloproteinases (MMPs), and connexin 37 (CX37) and 43 (CX43) have been shown to play important roles in the process of ECM remodeling and intercellular communication. A maternal treatment with N-acetyl-cysteine (NAC) or with an olive oil-supplemented diet was able to prevent alterations in the ECM remodeling process observed in the offspring’s heart of animal models of diabetes. An increased pro-oxidant/proinflammatory process and related molecules (VCAM-1, E-selectin, and NOX) have been shown to be involved in the cardiovascular alterations programmed by maternal diabetes. Maternal treatments with an olive oil-supplemented diet or with antioxidants, as idebenone or N-acetyl-cysteine have beneficial effects on this process. All these pathways are interconnected and the underlying mechanism affecting them may be epigenetic phenomena.

The mechanisms involved in fetal programming of heart damage in maternal diabetes reviewed in this study include the dysregulation of key players in cardiac cell metabolism, increased damaging effects of a pro-oxidant and proinflammatory environment, and alterations in the cardiac ECM remodeling and intercellular communication. As summarized in Figure 1, all these mechanisms and the main players that regulate these altered pathways are interconnected and differentially modulated by epigenetic modifications. This evidences the complex and multiple pathways able to induce programming of cardiovascular diseases in the offspring of diabetic dams, but also, due to the close interaction of these main pathways, brings opportunities to facilitate intervention to provide protective effects. Dietary treatments that improve the maternal metabolism altered by diabetes, such as those with probiotics or supplementation with olive or fish oil, have been shown to have beneficial effects in the cardiac cell metabolism of the offspring. Some of the studies reviewed in this work point to olive oil supplementation in the maternal diet and the maternal treatment with N-acetyl-cysteine as effective treatments able not only to improve cardiac cell metabolism but also to reverse the programmed damaging effect of the pro-oxidant and proinflammatory environment and the alterations in the cardiac ECM remodeling in the offspring heart. Further research addressing plausible treatments in animal models of maternal diabetes and their translation into clinical practice is encouraged.

## Author Contributions

RH and AJ designed the research. RH and ML conducted the research. RH wrote the manuscript. AJ contributed to the critical reading of the manuscript. All authors contributed to the article and approved the submitted version.

## Funding

This work was supported by the Agencia Nacional de Promoción Científica y Tecnológica de Argentina (PICT 2015 130 and PIDC 2015 0064).

## Conflict of Interest

The authors declare that the research was conducted in the absence of any commercial or financial relationships that could be construed as a potential conflict of interest.

## Publisher’s Note

All claims expressed in this article are solely those of the authors and do not necessarily represent those of their affiliated organizations, or those of the publisher, the editors and the reviewers. Any product that may be evaluated in this article, or claim that may be made by its manufacturer, is not guaranteed or endorsed by the publisher.

## Abbreviations

VCAM-1: Vascular cell adhesion molecule-1
GDM: Gestational diabetes mellitus
HUVEC: Human umbilical vein endothelial cells
EZH2β: Enhancer of zester homolog 2 – β
STZ: Streptozotocin
*Hspa1a*: *Heat shock protein 1a1*
NPR1: Natriuretic Peptide Receptor 1
PANK1: Panthothenate kinase
SCAND1: SCAN domain-containing protein 1
Sirt1: Silent information regulator 1
FoxO1: Forkhead box protein O1
FGF: Fibroblast growth factor
GSK3β: Glycogen synthase kinase 3 β
PGC1α: Peroxisome proliferator-activated receptor gamma coactivator alpha
mTOR: Mechanistic target of rapamycin
SGK1: Serum- and glucocorticoid-inducible kinase 1
PPAR: Peroxisome proliferator activated receptor
NOX: NADPH oxidases
TBARS: Thiobarbituric acid reactive substances
ECM: Extracellular matrix
TGFβ-1: Transforming growth factor β-1
CTGF: Connective tissue growth factor
